# Accuracy of fractal analysis and PI-RADS assessment of prostate magnetic resonance imaging for prediction of cancer grade groups: a clinical validation study

**DOI:** 10.1007/s00330-021-08358-y

**Published:** 2021-12-18

**Authors:** Florian Michallek, Henkjan Huisman, Bernd Hamm, Sefer Elezkurtaj, Andreas Maxeiner, Marc Dewey

**Affiliations:** 1 Department of Radiology, Charité – Universitätsmedizin Berlin, corporate member of Freie Universität Berlin and Humboldt- Universität zu Berlin, Charitéplatz 1, 10117 Berlin, Germany; 2 Department of Radiology, Radboud University Nijmegen Medical Centre, Nijmegen, Netherlands; 3 Institute of Pathology, Charité – Universitätsmedizin Berlin, corporate member of Freie Universität Berlin and Humboldt- Universität zu Berlin, Berlin, Germany; 4 Department of Urology, Charité – Universitätsmedizin Berlin, corporate member of Freie Universität Berlin and Humboldt- Universität zu Berlin, Berlin, Germany

**Keywords:** Prostatic neoplasms, Neoplasm grading, Perfusion, Fractals, Multiparametric magnetic resonance imaging

## Abstract

**Objectives:**

Multiparametric MRI with Prostate Imaging Reporting and Data System (PI-RADS) assessment is sensitive but not specific for detecting clinically significant prostate cancer. This study validates the diagnostic accuracy of the recently suggested fractal dimension (FD) of perfusion for detecting clinically significant cancer.

**Materials and methods:**

Routine clinical MR imaging data, acquired at 3 T without an endorectal coil including dynamic contrast-enhanced sequences, of 72 prostate cancer foci in 64 patients were analyzed. In-bore MRI-guided biopsy with International Society of Urological Pathology (ISUP) grading served as reference standard. Previously established FD cutoffs for predicting tumor grade were compared to measurements of the apparent diffusion coefficient (25th percentile, ADC_25_) and PI-RADS assessment with and without inclusion of the FD as separate criterion.

**Results:**

Fractal analysis allowed prediction of ISUP grade groups 1 to 4 but not 5, with high agreement to the reference standard (*κ*_FD_ = 0.88 [CI: 0.79–0.98]). Integrating fractal analysis into PI-RADS allowed a strong improvement in specificity and overall accuracy while maintaining high sensitivity for significant cancer detection (ISUP > 1; PI-RADS alone: sensitivity = 96%, specificity = 20%, area under the receiver operating curve [AUC] = 0.65; versus PI-RADS with fractal analysis: sensitivity = 95%, specificity = 88%, AUC = 0.92, *p* < 0.001). ADC_25_ only differentiated low-grade group 1 from pooled higher-grade groups 2–5 (*κ*_ADC_ = 0.36 [CI: 0.12–0.59]). Importantly, fractal analysis was significantly more reliable than ADC_25_ in predicting non-significant and clinically significant cancer (AUC_FD_ = 0.96 versus AUC_ADC_ = 0.75, *p* < 0.001). Diagnostic accuracy was not significantly affected by zone location.

**Conclusions:**

Fractal analysis is accurate in noninvasively predicting tumor grades in prostate cancer and adds independent information when implemented into PI-RADS assessment. This opens the opportunity to individually adjust biopsy priority and method in individual patients.

**Key Points:**

*• Fractal analysis of perfusion is accurate in noninvasively predicting tumor grades in prostate cancer using dynamic contrast-enhanced sequences (κ*_*FD*_ = *0.88).*

*• Including the fractal dimension into PI-RADS as a separate criterion improved specificity (from 20 to 88%) and overall accuracy (AUC from 0.86 to 0.96) while maintaining high sensitivity (96% versus 95%) for predicting clinically significant cancer.*

*• Fractal analysis was significantly more reliable than ADC*_*25*_* in predicting clinically significant cancer (AUC*_*FD*_ = *0.96 versus AUC*_*ADC*_ = *0.75).*

## Introduction

The grading of prostate cancer (PCa) is highly important for its clinical management and further prognosis. The standard diagnostic pathway still includes a digital rectal examination and prostate-specific antigen (PSA) levels. If suspicious, a consecutive biopsy is performed with histological grading based on the Gleason grading system [1, 2] and modifications by the International Society of Urological Pathology (ISUP) and WHO through introduction of the five ISUP grade groups [3–5]. Importantly, patients with ISUP grade group 1 lesions, or group 2 lesions with a low percentage (< 10%) of Gleason score 4, can be considered for active surveillance [6].

According to the literature, some biomarkers such as the prostate health index (PHI), the prostate cancer gene 3 (PCA3), or the four kallikrein (4 K) showed additive value in discriminating between aggressive and non-aggressive tumors and ISUP grade groups, respectively [7]. However, upfront multiparametric magnetic resonance imaging (mpMRI) at 3 T has good sensitivity and negative predictive value in detecting prostate cancer [8–10], including the transitional zone [11], and is implemented according to the Prostate Imaging Reporting and Data System version 2.1 (PI-RADS v2.1) [12]. Tracer kinetic parameters obtained from perfusion MRI [13] as well as the apparent diffusion coefficient (ADC) have shown some relation to tumor aggressiveness [14, 15]. However, no method allows noninvasive tumor grade prediction with clinically adequate accuracy.

**Fig. 1:**
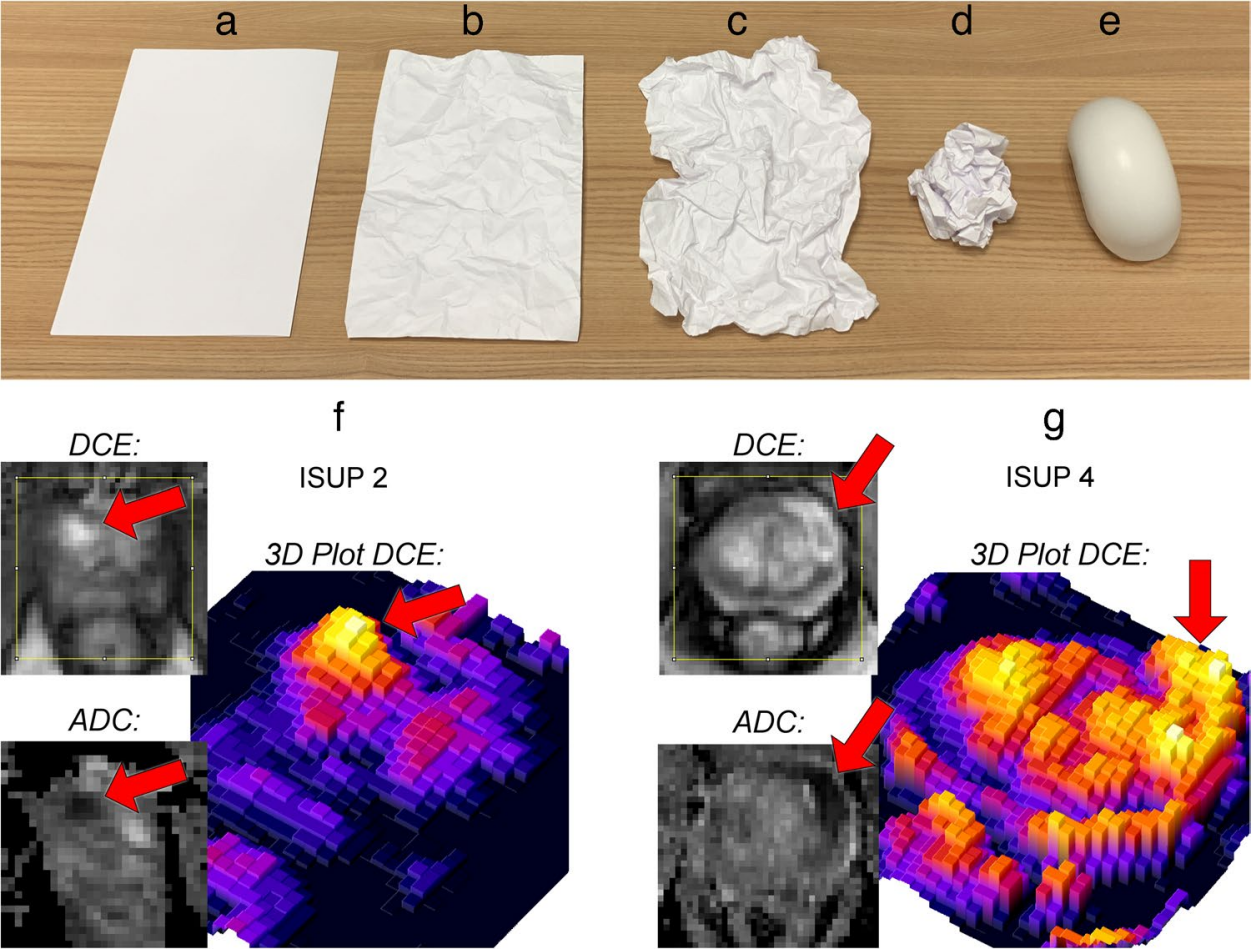
Significance of the fractal dimension (FD). The FD can be interpreted as a measure of complexity or chaos. Consider a sheet of paper, which is a two-dimensional object when neglecting its thickness (**a**). When the sheet is crumpled up, it occupies a certain volume and its geometrical complexity, or chaos, increases according to how much it is crumpled (**b–d**), representing an object with an FD between 2 and 3, until it becomes a three-dimensional object (**e**). Consequently, the dimensionality of the sheet, or its FD, exceeds its topological dimension of 2 (**a**) and is capped by the embedding dimension of 3 (**e**) with the actual value being somewhere in between. In this example, the FD measures the amount of crumpling, which represents a chaotic structural alteration. Two objects with a similar FD do not necessarily resemble each other. Rather, the FD can be considered a descriptor of the object’s geometrical complexity. In medical imaging, data can be represented in topologically two-dimensional grayscale images as in F and G, depicting a timepoint of dynamic contrast-enhanced (DCE)-MRI sequences with a prostate cancer focus (arrows, F: ISUP grade group = 2, G: group = 4) and the corresponding apparent diffusion coefficient (25th percentile, ADC_25_) map for correlation. These images can be interpreted as textures or terrain maps with intensity representing the texture’s height, as visualized in the colored 3D plots. Thus, such images can be assigned a topological dimension of 2 and an embedding dimension of 3, similar to the crumpled sheet of paper. An intensity distribution with a high spatial correlation, i.e., small amount of chaos or a well-defined transition, tends toward integer FD values (near 2.0 or 3.0), whereas randomly distributed intensity variations tend toward an FD of 2.5. The tumor margin in F (FD = 2.262, ISUP grade group = 2) is less chaotic than that in G (FD = 2.421, ISUP grade group = 4), which is reflected by the respective FD and can also be appraised visually in 3D. Because the FD integrates heterogeneity with spatial adjacency and correlation of signal intensity, fractal analysis constitutes a meaningful measure to quantify biological chaos in medical imaging

PCa can be separated into a tumor core compartment and a tumor margin. Angiogenesis in PCa mainly occurs in the margin, and the newly formed blood vessels show a more chaotic and dynamic organization compared to the more stabilized vasculature in the tumor core [16]. Thus, the organization of blood vessels in the margin and the resulting perfusion pattern might allow conclusions to be drawn on the degree of tumor differentiation. Branching patterns of the vascular tree are a multi-scale phenomenon and are known to have a fractal structure. Fractal geometry is a fundamental principle of biological structure and function with scale invariance as a pivotal characteristic. Perfusion, as a physiological process, features a fractal organization, which can be assessed by applying fractal analysis to images acquired by radiological and nuclear medicine imaging methods [17, 18]. Fractal analysis yields the fractal dimension (FD) as a quantitative measure of geometrical roughness or chaos (Fig. 1).

Both vascularity and perfusion characteristics of prostate cancer have been under debate in the past. Histological hypovascularity with a reduced microvascular density of cancer foci in comparison to non-cancerous prostate tissue has been suggested, depending on the employed counting methods [19]. Moreover, the role of dynamic contrast-enhanced (DCE) imaging is currently of minor priority for clinical management with a trend toward non-contrast biparametric protocols [20]. However, a common radiological observation is the often moderate to high affinity to contrast agent of cancer lesions especially in the early arterial phase, which indicates a comparatively low microvascular resistance. This observation—both visually and quantitatively by perfusion parameters, e.g., Ktrans [13]—has not been sufficiently consistent to justify DCE as a major PI-RADS criterion. However, it is conceivable that angiogenesis and perfusion, for being hallmarks of cancer, still hold important biological information and that the conventional methods of analysis simply do not provide sufficient insight. As we observed previously [21], perfusion chaos—which is quantified by fractal analysis—unveils information on the underlying vascular structure and can be related to tumor dedifferentiation. Therefore, we suggest fractal analysis as an alternative approach to perfusion imaging to access information that is implied in DCE sequences but has not yet been adequately assessed.

This study sought to validate the potential of previously established quantitative FD thresholds for their clinical application in routine MRI using an openly available dataset with in-bore MRI-guided biopsy as reference. We evaluated the implementation of fractal analysis into PI-RADS assessment as a separate criterion to examine its additional value in an integrated imaging workup for PCa detection and characterization and compared its performance with ADC measurements for peripheral and transitional-zone PCa.

## Materials and methods

### Patients and imaging dataset

In collaboration with Radboud University investigators, we retrospectively analyzed the testing cohort of the publicly available imaging dataset from the PROSTATEx2 challenge [21–23], which included clinical MRI examinations and nonpublicly revealed histological grading results. The original PROSTATEx2 challenge data were collected from a consecutive series of routine patients undergoing mpMRI due to clinical suspicion of prostate cancer with elevated PSA levels (> 4 ng/ml) or abnormal digital rectal examination findings. No restrictions on prior biopsies were imposed. In our retrospective study, we included all patients from the PROSTATEx2 dataset with malignant histology in at least one of the reported lesions and excluded any patients in whom malignancy was not histologically proven. To enable a separate analysis of PCa in the transitional zone, an eligible subgroup was identified which additionally featured patients from the training cohort. The dataset was openly published under the Creative Commons Attribution 3.0 Unported License. The ISUP grade groups were determined from in-bore MRI-guided biopsy and served as reference standard. The location of PCa foci is available in terms of image coordinates to ensure consistency in lesion identification. Details of the imaging protocol are provided in the original dataset publication [21] and are summarized in Table 1. In brief, two different 3-T MRI scanners from the same manufacturer were used, and the imaging protocol included T2-weighted, proton density-weighted, dynamic contrast-enhanced (DCE), and diffusion-weighted imaging (DWI) with ADC mapping. DCE images were acquired with a 3D turbo flash gradient echo sequence (in-plane resolution of around 1.5 mm, 4 mm slice thickness, 3.5 s temporal resolution) with intravenous administration of a gadolinium-based contrast agent. Direct, in-bore endorectal biopsies were performed with as many cores as deemed necessary by the number of lesions detected on mpMRI. Typically, two cores per suspicious lesion were sampled and the procedure usually took around 45–60 min. Histological analysis was performed by an experienced (> 20 years) uropathologist. Data used in this research were obtained from The Cancer Imaging Archive (TCIA) sponsored by the international society for optics and photonics (SPIE), National Cancer Institute/National Institutes of Health (NCI/NIH), American Association of Physicists in Medicine (AAPM), and Radboud University [24].

**Table 1:** Magnetic resonance imaging protocol

Parameter	T1-weighted DCE sequence	T2-weighted sequence	Diffusion-weighted imaging	Apparent diffusion coefficient
Field strength (all sequences)	3 T			
Coil (all sequences)	Pelvic phased-array coil without endorectal coil			
Fat suppression	No			
Pulse sequence	3D turbo flash gradient echo	2D turbo spin echo	Single-shot echo-planar imaging (three directions)	Secondary calculation
Orientations	Axial	Axial, sagittal, coronal	Axial	Axial
Resolution (mm)	1.5 × 1.5	~ 0.5 × 0.5	2 × 2	2 × 2
Slice thickness (mm)	4	3.6	3.6	3.6
Field of view (mm)	192 × 192	180 × 180–192 × 192	168 × 256	168 × 256
Temporal resolution	Every 3.5 s for 2:40 min	n.a	n.a	n.a
*b* values (s/mm^2^)	n.a	n.a	Measured: 50, 400, 800 calculated: 1400	n.a

*DCE* dynamic contrast enhanced, *n.a.* not applicable

### Fractal analysis

The FD, as the quantitative result of fractal analysis, constitutes a meaningful measure of biological chaos. An illustrative explanation can be found in Fig. 1, and a comprehensive review of fractal analysis of perfusion imaging is provided by Michallek and Dewey [17]. As both the anatomical vascular structure and function are fractal, the phenotype of the perfusion pattern depends on the underlying vascular tree and its architecture is scale-invariant. Therefore, changes in the FD of the perfusion pattern directly convert to changes in the vascular tree. To quantify the chaos of the perfusion pattern, fractal analysis was applied to DCE-MRI sequences. The local FD was calculated [25] based on the fractal blanket dimension [26], which evaluates feature propagation over multiple scales. This can be realized in terms of a blanket that is molded to the texture. The blanket is iteratively raised from the texture thereby losing detail. The FD can be obtained from quantifying the loss of detail as a function of distance between the iteratively raised blanket and the original texture. A bi-logarithmic linear regression of observed feature against scale is performed with the slope determining the FD. A visual introduction to fractal analysis and its significance is shown in Fig. 1 with an example of a crumpled sheet of paper and prostate MRI texture. From the whole DCE time series, we extracted the maximum FD of the tumor margin, which was subjected to statistical analysis. Typically, the maximum FD is achieved during the early phase of contrast enhancement, which is hypothesized to reflect differences in vascular architecture between hyperperfused tumor tissue and adjacent tissue. This study validates the FD cutoffs for individual ISUP grade groups as established in [21]. The analysis was performed by one reader (6 years of experience in urogenital imaging and prostate MRI) and was repeated in a subcohort of 50 lesions by an independent senior reader with > 15 years of experience in urogenital imaging and prostate MRI to assess interreader variability. Moreover, the junior reader repeated analysis of 25 lesions to assess intrareader variability.

### Image processing

**Fig. 2:**
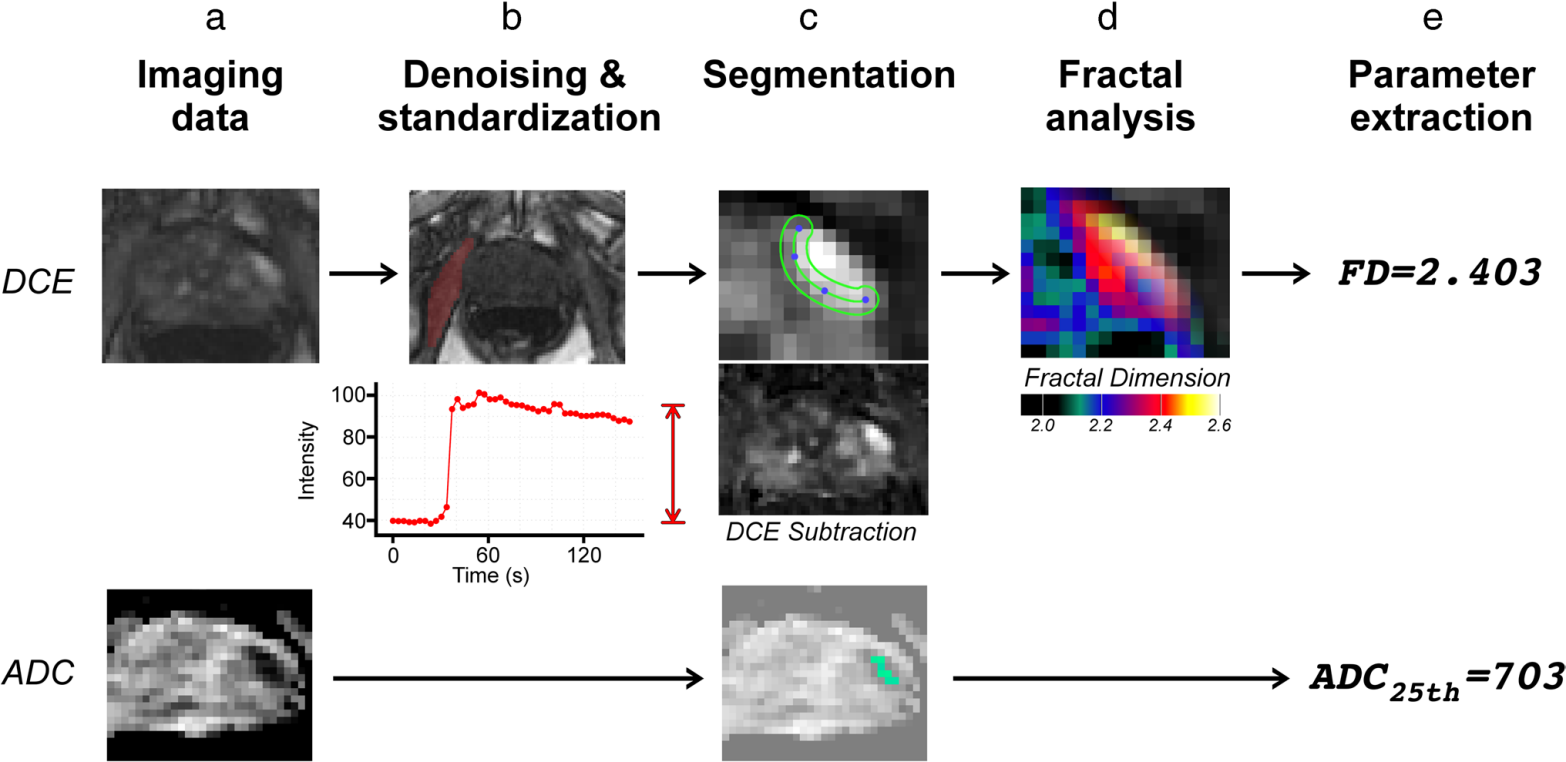
Image processing pipeline. The cancer foci have been visually correlated on all multiparametric sequences including the dynamic contrast-enhanced (DCE) sequence, ADC maps, T2-weighted (T2w) sequence, and diffusion-weighted images (DWI and T2w not shown) (**a**). A denoising and intensity standardization scheme was applied to the DCE images, which includes intensity measurement in the internal obturator muscle (dark red ROI and diagram) with extraction of the mean and standard deviation in unenhanced and contrast-enhanced phases (**b**). Subsequently for fractal analysis, the tumor margin on DCE images was segmented in a standardized, semi-automatic manner to comprise the hyperperfused tumor periphery in adjacency to surrounding prostate tissue. Any peripheral tumor parts with contact to the prostate capsule was not included in the segmentation due to missing adjacent prostate tissue. For ADC measurements, the hotspot with the lowest ADC values was segmented (**c**). For DCE images, fractal analysis yields maps of fractal dimension (FD)(**d**). Finally, FD and ADC_25_ (25th percentile), were extracted and subjected to statistical analysis (**e**). In this example, an ISUP grade group 4 tumor is shown, which was correctly predicted by FD = 2.403. ADC_25_ was 703 × 10^−6^ mm^2^/s

The analysis procedure consisted of preprocessing, calculation of local FD maps, definition of the region of interest (ROI), and evaluation of fractal analysis results. During preprocessing, intensity was linearly calibrated according to the signal intensity of the internal obturator muscle before and after contrast administration. Image noise was estimated from the standard deviation of the pre-contrast signal intensity of the internal obturator muscle, and a bilateral filter was applied [27]. A paramedian slice location in relation to the tumor center was selected to obtain a preferably large depiction of the hypervascularized part of the tumor margin. From this slice location, two-dimensional maps of the local FD were calculated from the DCE image sequence for each point in time using a 3 × 3 pixel kernel. To evaluate the results of fractal analysis, a region of interest (ROI) was segmented containing the tumor margin, which was defined as the interface region between the hyperperfused part of the tumor and the adjacent tumor harboring prostate tissue. Segmentation was performed in a standardized, semi-automatic manner by fitting a serpentine-like region with a fixed width of 3 mm to the border of the hyperperfused tumor part as illustrated in Fig. 2. The respective ROI was defined in the DCE images and propagated to the local FD map at each point in time. The mean FD of the ROI was calculated and plotted over time with the ROI being adjusted in case of motion artifacts. The highest mean FD in the time sequence gives the maximum geometrical complexity of the perfusion pattern, which constitutes the pathophysiological relevance of the FD and was subjected to statistical analysis.

### ADC measurement

In addition to fractal analysis, the apparent diffusion coefficient (ADC) was measured for each PCa lesion. A freehand drawing tool was used to place regions of interest (ROIs) comprising the core of the tumor lesion with the lowest ADC and avoiding partial volume at the PCa border. No preprocessing other than segmentation was applied to ADC images. We performed ADC measurements by delineating the tumor region with the most marked diffusion restriction, i.e., the hotspot with the lowest ADC value, and we extracted the 25th percentile (ADC_25_, expressed as 10^−6^ mm^2^/s). An example delineation is shown in Fig. 2 (bottom row). We opted for this approach due to growing evidence that measuring lower percentiles of ADC in those hotspots improves correlation with tumor grade [21, 28–30]. The process is depicted in Fig. 2.

### PI-RADS v2.1 assessment

PI-RADS v2.1 assessment [12] was performed for each PCa lesion by an experienced reader (> 20 years experience in genitourinary imaging and prostate MRI), who was blinded to ISUP grading, fractal analysis results, and quantitative ADC_25_ measurements.

### Statistical analysis

Descriptive statistics and linear modeling were used to evaluate the correlation of FD, or ADC_25_, with ISUP grade groups. The Kruskal–Wallis test and pairwise group comparisons using the Mann–Whitney *U* test were performed to evaluate groupwise differences. Diagnostic accuracy was analyzed in terms of sensitivity and specificity. Moreover, agreement of FD, or ADC_25_, with ISUP grade groups was evaluated using quadratic-weighted kappa-statistics as in [23]. Receiver operating characteristics (ROC) analysis with area under the curve (AUC) calculation and cutoff determination by maximizing Youden’s *J* were performed for ADC_25_. Subgroup analysis of transitional-zone PCa was performed in a similar manner including a groupwise comparison with nontransitional-zone PCa using the Kruskal–Wallis test. Inter- and intrareader variability were assessed in terms of agreement using Cohen’s *κ* and Bland–Altman analysis. A level of *p* ≤ 0.05 was considered statistically significant, and adjusted *p* values with Bonferroni correction (where appropriate) are reported. The STARD guidelines were adhered to. Statistical analysis was performed with R (v3.4.1; 30 June 2017, R Foundation for Statistical Computing).

## Results

### Patient cohort

**Fig. 3:**
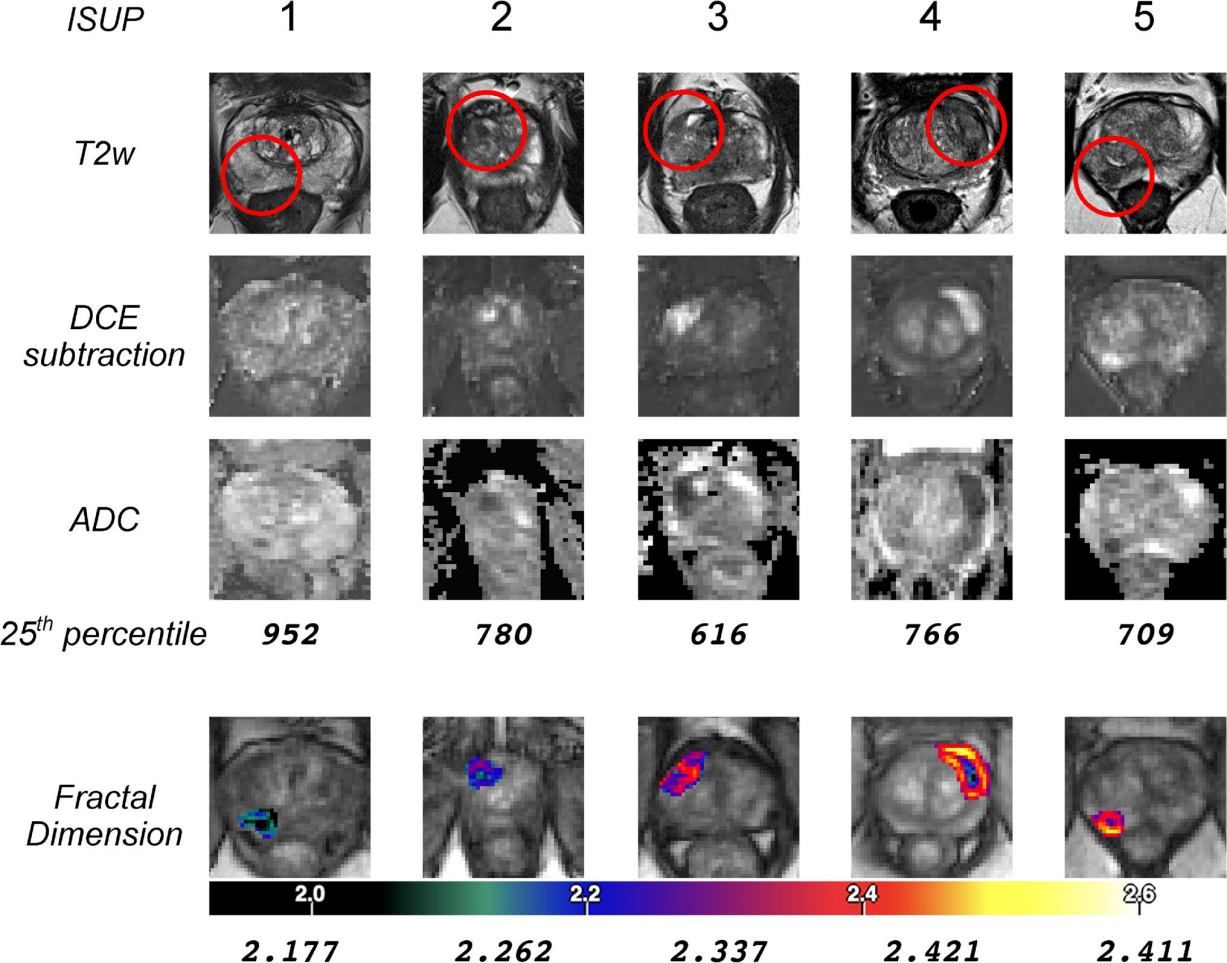
Example cases of fractal analysis. The multiparametric sequences (T2-weighted, T2w; dynamic contrast-enhanced, DCE; apparent diffusion coefficient, ADC) are shown with the fractal dimension (FD) map of the tumor. The quantitative values for ADC_25_ (25th percentile) or FD are given underneath the respective row. DCE subtraction depicts a phase during early first-pass and was standardized by baseline signal intensity

The patient cohort included 64 patients of the PROSTATEx2 challenge [23] with a total of 72 PCa lesions. The included patients had a median age of 66 years (range: 48–77 years), a median PSA level of 14 ng/ml (interquartile range: 12.5 ng/ml), and median lesion size of 18 mm (range: 8–36 mm). ISUP grade group distribution was as follows: ISUP 1 *n* = 23, ISUP 2 *n* = 26, ISUP 3 *n* = 9, ISUP 4 *n* = 8, and ISUP 5 *n* = 6. Fractal analysis was successfully performed in all lesions with a processing time of approx. 10 min per lesion (including denoising, intensity standardization, semi-automatic lesion segmentation, fractal analysis with generation of FD maps, and assessment of fractal analysis results). Examples are depicted in Fig. 3.

### Fractal analysis

**Fig. 4:**
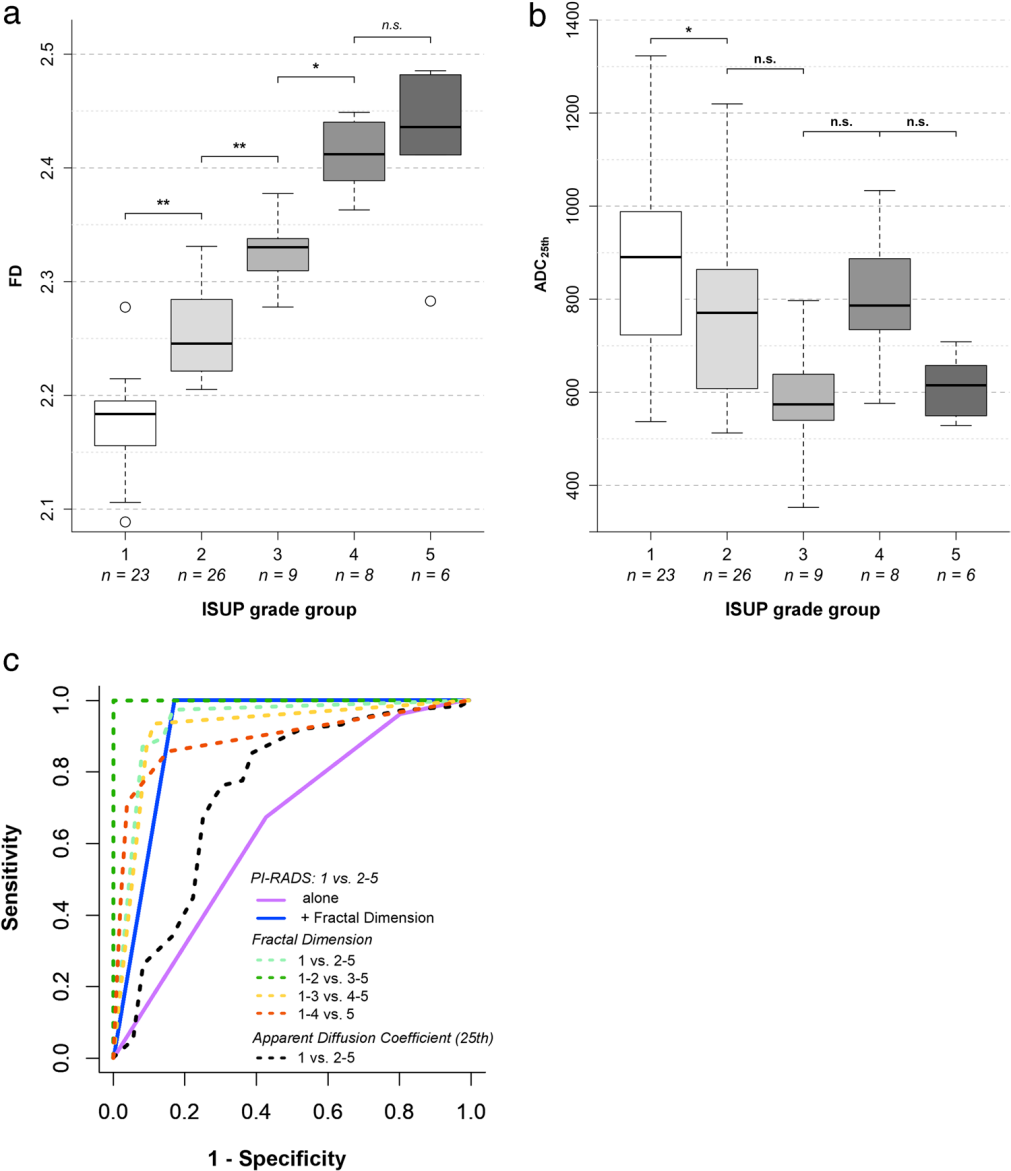
Results of the clinical validation. (**a**) Fractal dimension (FD) and (**b**) apparent diffusion coefficient (25th percentile, ADC_25_) against the ISUP grade group. ADC_25_ values are expressed as 10^−6^ mm^2^/s. (**c**) Receiver operating characteristic curves (ROC) with area under the curve (AUC) in the discovery cohort. Different ROC curves are shown: PI-RADS alone and in combination with FD to differentiate clinically significant and non-significant cancer lesions; FD to differentiate lesions in dichotomized pooled ISUP grade groups; ADC_25_ to differentiate clinically significant and non-significant lesions. *p < 0.005; **p < 0.001; n.s., not significant; *n*, sample size per group; 25th–25th percentile; ISUP grade group 1—Gleason score ≤ 6; group 2—Gleason score 3 + 4 = 7; group 3—Gleason score 4 + 3 = 7; group 4—Gleason score 4 + 4 = 8; 3 + 5 = 8; 5 + 3 = 8; group 5—Gleason scores 9–10

FD thresholds previously established in [21] were used in this study and are compiled in Table 2. Boxplots of FD and ADC_25_ for ISUP grade groups are shown in Fig. 4(a and b). Significant differences in FD were found for all pairwise grade group comparisons (*p* < 0.005), except for the highest ISUP grade groups (group 4 [*n* = 8], versus 5 [*n* = 6]). The previously established FD cutoffs showed very good performance (sensitivities, specificities, and confidence intervals given in Table 2), and FD was linearly correlated with grade group (*r*^2^ = 0.840, *p* < 0.001). Inter- and intrareader variability analysis showed high agreement (interreader *κ* = 0.89, CI: 0.82–0.95; intrareader *κ* = 0.96, CI: 0.91–1.0) without substantial bias (interreader: 0.02, intrareader: 0.001) and acceptable limits of agreement (interreader: − 0.03 to 0.06, intrareader: − 0.01 to 0.02).

**Table 2:** Evaluation of diagnostic performance of fractal analysis and apparent diffusion coefficient (25th percentile, ADC_25_) measurement using previously established cutoffs for fractal dimension (FD). Relative and absolute sensitivity and specificity for prediction of pooled ISUP grade groups are given with 95% confidence intervals (CI, in brackets). Sensitivity was defined as the fraction of correctly predicted lesions in the higher-grade group pool. The ADC_25_ cutoff is given in units of 10^−6^ mm^2^/s and has been established in this study

Pooled ISUP grade group comparison	AUC	Cutoff	Sensitivity	Specificity	*κ*
*Fractal dimension*					0.88 (CI: 0.79–0.98)
1 versus 2–5	0.96 (CI: 0.93–0.99)	2.20	100% (CI: 93–100%) 49/49	91% (CI: 72–99%) 21/23	
1–2 versus 3–5	0.99 (CI: 0.98–1.0)	2.31	83% (CI: 61–95%) 19/23	98% (CI: 89–100%) 48/49	
1–3 versus 4–5	0.98 (CI: 0.96–1.0)	2.40	79% CI: 49–95%) 11/14	100% (CI: 94–100%) 58/58	
*Apparent diffusion coefficient (25th percentile)*					0.36 (CI: 0.12–0.59)
1 versus 2–5	0.75 (CI: 0.68–0.83)	905	86% (CI: 73–94%) 42/49	48% (CI: 27–69%) 11/23	

*κ* quadratic-weighted kappa statistic

### PI-RADS v2.1 assessment

Using PI-RADS alone, assessment category ≥ 4 alone was highly sensitive (96%, CI: 91–99%) but not specific (20%, CI: 11–33%) for detecting clinically significant PCA. Implementing the FD as an independent criterion to PI-RADS assessment, sensitivity was maintained (95%, CI: 90–98%) while significantly improving on specificity (88%, CI: 77–95%) and AUC (PI-RADS with FD AUC = 0.92, CI: 0.87–0.96, vs. PI-RADS alone AUC = 0.65, CI: 0.57–0.73, *p* < 0.001; Fig. 4(c)).

### Comparison with ADC measurements

ADC_25_ showed a moderate linear correlation (*r*^2^ = 0.253, *p* < 0.001) and a moderate performance for differentiating ISUP grade group 1 versus pooled groups 2–5 only (AUC_ADC_ = 0.75, CI: 0.68–0.83), but no significant differences between the other grade groups.

Agreement with ISUP grade groups was higher for fractal analysis than for ADC_25_ with quadratic-weighted kappa values *κ*_FD_ = 0.88 (CI: 0.79–0.98) for FD in a multi-class prediction (individual groups 1–5) and *κ*_ADC_ = 0.36 (CI: 0.12–0.59) for ADC_25_ in single-class prediction of group 1 versus groups 2–5 (only significantly different comparison for ADC_25_).

### Transitional-zone subgroup analysis

**Fig. 5:**
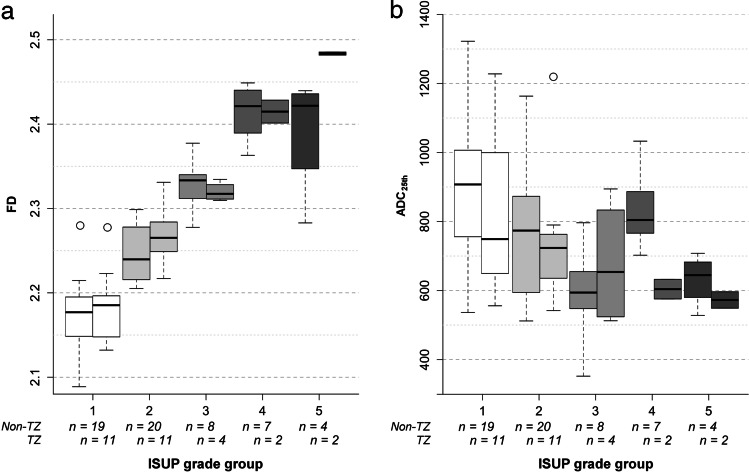
Results of the subgroup analysis per prostate carcinoma (PCa) location for fractal analysis (**a**) and apparent diffusion coefficient measurement (25th percentile, ADC_25_) (**b**). Intra-group analysis did not show significant differences by focus location for FD (**a**) and minor differences for ISUP grade group 4 in ADC_25_ measurement (**b**), which might be interpreted as outliers with *n* = 2 for transitional-zone group 4 PCa. FD—fractal dimension, ADC_25_—25th percentile of apparent diffusion coefficient in 10^−6^ mm^2^/s, TZ—transitional zone

For subgroup analysis of transitional-zone PCa, the 16 patients from the PROSTATEx2 dataset were supplemented by 14 eligible patients from the testing cohort in the PROSTATEx dataset, yielding 30 patients with 30 transitional-zone PCa foci. Results of this group analysis are summarized in Fig. 5. Importantly, no significant differences were found between transitional and peripheral zone PCa (*p* ranging from 0.06 to 0.77).

## Discussion

Fractal analysis of perfusion MRI showed clinically reasonable performance for noninvasively differentiating low-, intermediate-, and high-grade prostate cancer (corresponding to ISUP grade groups 1–4) and significantly improved specificity of PI-RADS assessment for detecting clinically significant cancer. Previously established FD thresholds for the different ISUP grade groups seem to be readily applicable in a clinical context when using DCE-MRI sequences. Fractal analysis was robust regardless of the anatomic prostate zone in which the PCa focus was localized and had comparable diagnostic performance in a subgroup analysis of transitional-zone PCa. Additionally, our results indicate that fractal analysis might be more accurate than ADC_25_ measurement for tumor grade prediction.

Both the vascularity and the perfusion characteristics of prostate cancer have been debated in the past, and the role of DCE imaging is currently of minor priority for clinical management. However, a common radiological observation is the often moderate to high affinity to contrast agent of cancer lesions especially in the early arterial phase, which indicates a comparatively low microvascular resistance. This observation—both visually and quantitatively by perfusion parameters, e.g., Ktrans—has not been sufficiently consistent to justify DCE as a major PI-RADS criterion. However, it is conceivable that angiogenesis and perfusion, for being hallmarks of cancer, still hold important biological information and that the conventional methods of analysis do not provide sufficient insight. This was a major motivation for our study. As we observed previously [21], perfusion chaos—which is quantified by fractal analysis—unveils information on the underlying vascular structure and can be related to tumor dedifferentiation. Therefore, we suggest fractal analysis as an alternative approach to perfusion imaging to access the information being implied in DCE sequences but having not yet been adequately assessed by conventional methods. In light of existing clinical prostate imaging protocols, our findings suggest that DCE-MRI sequences have added value for comprehensive pathophysiological analysis of perfusion patterns.

There are several clinical circumstances in which fractal analysis of DCE sequences might improve clinical management of patients with prostate cancer: In the subpopulation of patients with equivocal likelihood of clinically significant cancer and PI-RADS 3 lesions, fractal analysis might noninvasively help to decide on biopsy priority and method as well as assignment to active surveillance. Moreover, fractal analysis might constitute a parameter of progression in patients under active surveillance. In high-risk patients with PI-RADS 4 and 5 lesions, fractal analysis might allow differentiation of high-grade cancer, thus streamlining clinical management. Another potential application could be treatment monitoring in patients undergoing radiotherapy or prostate embolization.

### Angiogenesis in prostate cancer

Angiogenesis plays an important role in the development of PCa [31]. Especially in the tumor margin, development of new blood vessels is highly dynamic and results in a chaotic architecture, while a more orderly vascular pattern is observed in the tumor center [16, 32]. This phenomenon has been related to pericyte density, which was found to be lower in the tumor margin and results in an immature and chaotic phenotype, which is more pronounced in tumors with higher Gleason scores [33]. The relevance of angiogenesis and perfusion for tumor development and its correlation with the tumor grade has been shown in various studies of contrast kinetic parameters [13, 34–36] and microvascular architecture [32, 37–39]. Moreover, vascular morphology has been identified as an independent predictor of clinical outcome due to its relevance for tumor progression and metastatic potential [36]. In this study, we investigated the perfusion pattern in the tumor margin using fractal analysis, which can quantify the chaos of perfusion patterns and relate it to the underlying vascular structure. Thereby, fractal analysis captures and integrates architectural alterations of vessel morphology [40–42], tumor-specific peculiarities such as vasculogenic mimicry [43], tissue composition, and tumor sparsity [44, 45] as well as differences in vascularization between tumor and normal prostate tissue [46].

### Pathophysiological implications of fractal analysis

The FD was continuously distributed across ISUP grade groups 1 to 4, which might reflect a continuously increasing vascular dedifferentiation with increasing tumor grade but not the discrete boundaries separating individual grade groups. The Gleason grading system has traditionally been used for clinical decision-making and constitutes a decent estimator of the patient’s prognosis. However, along with the emergence of more elaborate imaging techniques and clinical evidence of the diagnostic performance of multiparametric MRI [8, 9] in conjunction with the recently published PI-RADS version 2.1 [12], a pathophysiologically comprehensive method for prediction of tumor grade has become desirable. Despite an ongoing trend toward ever-shorter imaging protocols [47], no reliable method has been clinically implemented to noninvasively predict PCa aggressiveness. Measuring ADC, especially lower percentiles, has been suggested as a method to differentiate low-grade PCa, i.e., ISUP grade group 1, from intermediate- and high-grade PCa, i.e., grade groups 2–5, and the diagnostic performance of ADC_25_ measurement in our study confirms earlier results [14, 15, 21, 28–30, 48]. Our results indicate that ADC_25_ is inferior to fractal analysis of perfusion, which, in addition to better differentiation of indolent from significant cancer, allows accurate stratification of individual PCa foci according to their histological grade based on apparently pathognomonic perfusion patterns. This finding gains relevance in light of the WHO’s recent recommendation to pathologists to report the fraction of Gleason grade 4 and to consider reporting of the Gleason grade 5 fraction [5]. On the one hand, the precise characterization of intermediate-grade lesions has important prognostic implications [49–51]. On the other hand, patients with ISUP grade group 2 lesions and a low Gleason grade 4 fraction might be eligible for active surveillance [6].

### Use of contrast agent

Our preprocessing scheme, specifically the individual denoising and intensity standardization protocols, enables us to dynamically account for individual differences in noise and contrast. Therefore, the contrast agent dose is not expected to introduce a major bias. This aspect is relevant when implementing a low-contrast dose protocol as by He et al. [52]. Moreover, it might be possible to abbreviate the DCE-MRI protocol to only capture the first-pass phase, during which the peak FD was usually found. Abbreviating the DCE protocol and using a low amount of contrast agent might be an alternative approach to entirely skipping the DCE sequences, which constitutes a recent trend in prostate MRI and is also reflected in the recent PI-RADS version 2.1 [12].

### Limitations

Our study has limitations. Clinical patient data such as stage, clinical management, or follow-up findings were not included in the available retrospective dataset we used in our study. Therefore, our results allow no conclusions to be drawn regarding the potential of fractal analysis to predict clinical outcomes. The scope of fractal analysis of perfusion was characterizing rather than detecting suspicious lesions, for which PI-RADS is validated. Since we retrospectively analyzed an openly available dataset, we confined our analysis to the reported malignant lesions without having follow-up information available. However, fractal analysis—as investigated in our study—relies on the identification of a lesion through PI-RADS. Therefore, our reference standard refers to the per-lesion level, for which in-bore MRI biopsy has shown high accuracy including difficult and small cancer lesions (e.g., [53]). We do not expect the presence of follow-up information to substantially confound the performance of fractal analysis. While the minimum lesion size in this study was 8 mm, the majority of lesions was over 1 cm in diameter; therefore, an analysis of subcentrimetric lesions in a dedicated dataset might be insightful. Moreover, no information on the histological PCa subtype was available, which may gain relevance given the introduction of intraductal carcinoma as a new entity of PCa as well as new variants of acinar PCa into the classification of tumors by the WHO [5]. Histological characteristics and tumor entity are likely to affect prognosis or diagnostic accuracy. Therefore, dedicated fractal analysis of perfusion according to histological tumor characteristics might be insightful.

### Conclusion

In conclusion, fractal analysis of prostate perfusion improves the diagnostic accuracy of mpMRI for detecting clinically significant cancer and complements PI-RADS assessment toward an integrated imaging workup for PCa detection and characterization. The FD fosters the concept of quantitative imaging based on a valid pathophysiological model.

## Abbreviations

DCE: Dynamic contrast-enhanced
FD: Fractal dimension
ISUP: International Society of Urological Pathology
mpMRI: Multiparametric MRI
PCa: Prostate carcinoma

## Declarations

### Guarantor

The scientific guarantor of this publication is Professor Marc Dewey.

### Statistics and Biometry

Alejandra Rodríguez Sánchez kindly provided statistical advice for this manuscript.

### Methodology

• retrospective

• experimental

• data from one center, analysis at two centers
